# Development and Patient-Level Internal Validation of an MRI-Based Radiomics Machine Learning Model for Preoperative Prediction of Cervical Lymph Node Metastasis in Oral Squamous Cell Carcinoma: A Pilot Study

**DOI:** 10.3390/diagnostics16193220

**Published:** 2026-10-05

**Authors:** İrem Öztürk, Ferhat Erişir, Kaan Orhan, Gürkan Ünsal

**Affiliations:** 1 Department of Dentomaxillofacial Radiology, Faculty of Dentistry, Ankara University, 06560 Ankara, Türkiye; irmozturk@ankara.edu.tr (İ.Ö.); knorhan@dentistry.ankara.edu.tr (K.O.); 2 Department of Otorhinolaryngology and Head and Neck Surgery, Faculty of Medicine, Near East University, 99138 Kyrenia, Cyprus; ferhat.erisir@neu.edu.tr; 3 Schulich School of Medicine and Dentistry, Western University, London, ON N6A 5C1, Canada

**Keywords:** lymph node metastasis, oral squamous cell carcinoma, magnetic resonance imaging, radiomics, machine learning, model validation, class imbalance

## Abstract

**Background**/**Objectives**: Cervical lymph node metastasis (LNM) is the main prognostic factor in oral squamous cell carcinoma (OSCC) and guides the extent of neck treatment. This retrospective pilot study aimed to develop MRI-based radiomics machine learning models for the preoperative prediction of LNM in OSCC and to validate them internally at the patient level. **Methods**: Preoperative MRI of 74 patients (32 LNM-positive, 42 LNM-negative on histopathology) was analysed. In each patient the largest morphologically suspicious cervical lymph node was segmented volumetrically on axial contrast-enhanced T1-weighted images, and 1409 IBSI-compliant radiomic features were extracted. Performance was estimated by 10 repeats of 5-fold nested cross-validation with the patient as the unit of partitioning; standardisation, correlation pruning, LASSO selection, hyperparameter tuning and threshold choice were confined to the training folds. Six classifiers were compared. **Results**: LASSO retained a median of nine features (range 6–13). SVM performed best, with an AUC of 0.781 (95% CI 0.667–0.884), a sensitivity of 0.72, a specificity of 0.76 and a balanced accuracy of 0.74; XGBoost (AUC 0.774) and logistic regression (AUC 0.769) performed comparably. A slice-level analysis of the same data, in which slices from the same patient could fall into both training and test sets, gave an apparent AUC of 0.878 but a specificity of only 0.33. **Conclusions**: MRI radiomic features of cervical lymph nodes carry moderate patient-level information on LNM in OSCC, and slice-level designs can substantially misrepresent performance. External validation in larger multicentre cohorts is required before clinical use.

## 1. Introduction

Cancers of the lip and oral cavity are among the most frequent malignancies of the head and neck region. GLOBOCAN estimated approximately 390,000 new cases and 188,000 deaths from lip and oral cavity cancer worldwide in 2022, and more than 90% of these tumours are squamous cell carcinomas [1,2]. Cervical lymph node metastasis (LNM) is the most important prognostic factor in oral squamous cell carcinoma (OSCC) and determines the extent of neck treatment [3,4].

Magnetic resonance imaging (MRI) complements clinical examination and is widely used as an adjunctive diagnostic modality. Although MRI reliably evaluates major prognostic features such as tumour size and depth of invasion, its performance in detecting nodal metastasis is limited, especially for small and non-enlarged nodes. Meta-analyses restricted to head and neck squamous cell carcinomas have reported only moderate pooled sensitivity and specificity for MRI, with a further fall in sensitivity in the clinically node-negative (cN0) neck [5,6]. Evidence suggests that radiomic analysis of the primary tumour or of the nodes themselves may improve the preoperative prediction of lymph node metastasis across several tumour types [7,8,9,10,11].

Radiomics converts medical images into high-dimensional quantitative data that can be analysed with machine learning [12,13]; texture analysis in particular characterises the spatial distribution of grey levels and signal intensity beyond what visual inspection allows [14,15,16]. Nevertheless, relatively few studies have applied radiomics to nodal staging in OSCC.

The aim of this pilot study was to develop MRI-based radiomics machine learning models for the preoperative prediction of cervical LNM in OSCC and to validate them internally at patient level, using repeated nested cross-validation with all data-dependent steps confined to the training data.

## 2. Materials and Methods

This retrospective pilot study was conducted in accordance with the Declaration of Helsinki and approved by the Non-Interventional Clinical Research Ethics Committee of Lokman Hekim University (Protocol No: 2024/56, 23 February 2024). Only anonymised clinical and imaging data were used. Radiomic feature definitions follow the Image Biomarker Standardisation Initiative (IBSI) [17], and the study is reported according to the Checklist for Artificial Intelligence in Medical Imaging (CLAIM) [18] and the TRIPOD+AI statement [19].

### 2.1. Patients

**Figure 1:**
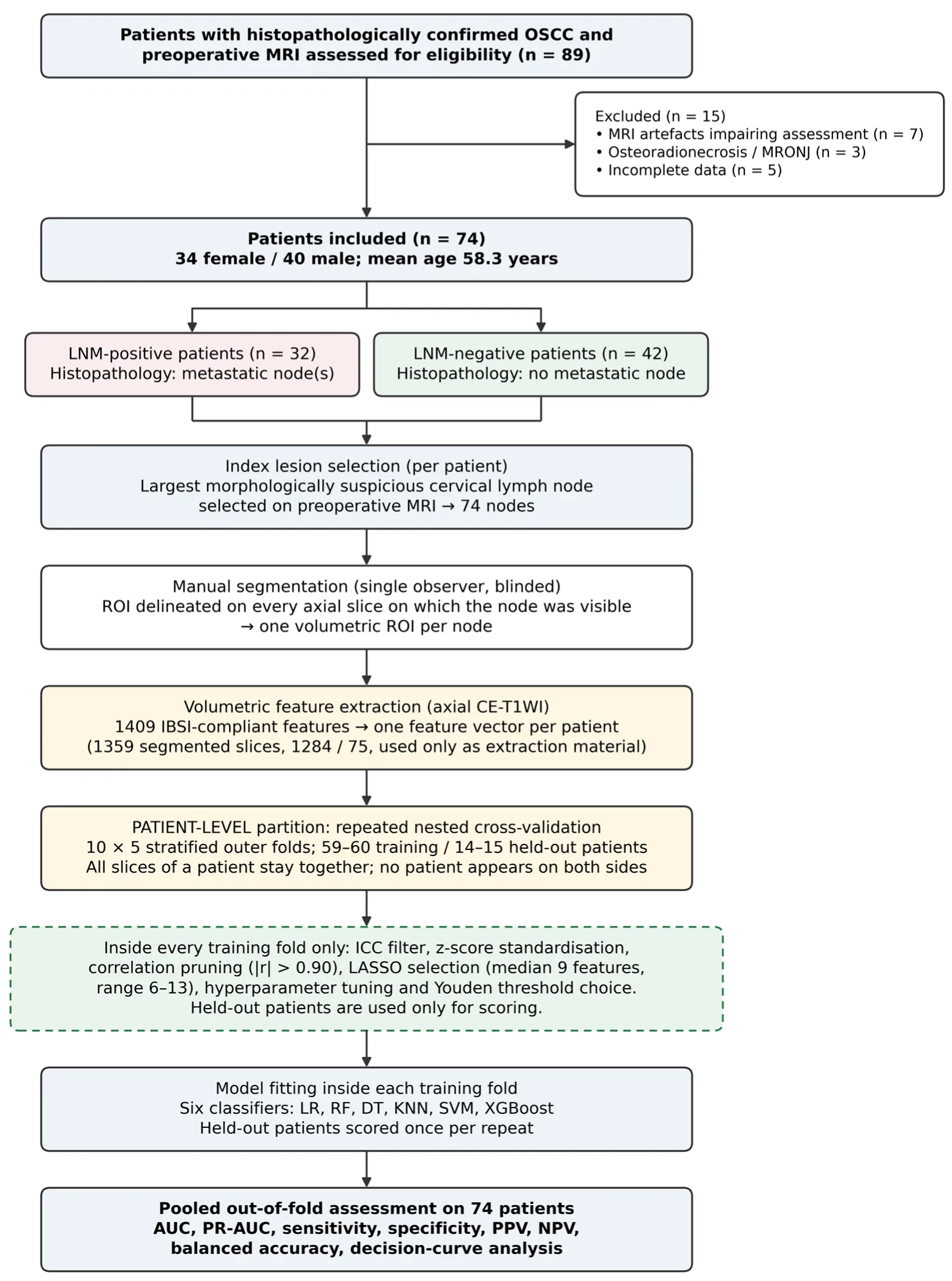
Study flow diagram: patient selection, segmentation, volumetric feature extraction and the patient-level nested cross-validation scheme. The dashed box marks the steps performed within each training fold. OSCC: oral squamous cell carcinoma; MRI: magnetic resonance imaging; MRONJ: medication-related osteonecrosis of the jaw; LNM: lymph node metastasis; ROI: region of interest; CE-T1WI: contrast-enhanced T1-weighted imaging; IBSI: Image Biomarker Standardisation Initiative; ICC: intraclass correlation coefficient; LASSO: least absolute shrinkage and selection operator; LR: logistic regression; RF: random forest; DT: decision tree; KNN: k-nearest neighbours; SVM: support vector machine; XGBoost: extreme gradient boosting; AUC: area under the receiver operating characteristic curve; PR-AUC: area under the precision–recall curve; PPV/NPV: positive/negative predictive value.

Patients aged 18 years or older with histopathologically confirmed OSCC and preoperative MRI were eligible. Patients were excluded if MRI showed significant artefacts impairing assessment, if they had pre-existing osteoradionecrosis or medication-related osteonecrosis of the jaw, or if histopathological confirmation or follow-up data were missing. Seventy-four patients (34 women, 40 men; mean age 58.3 years, range 45–74) were included, of whom 32 were LNM-positive and 42 LNM-negative on histopathology (Figure 1).

### 2.2. Reference Standard

The reference standard was histopathological examination of the neck dissection specimen. The node selected as the imaging target was marked on preoperative MRI, communicated to the operating surgeon, and retrieved and examined as a separately labelled specimen, so that imaging and pathology correspond at node level. A patient was classified as LNM-positive if the segmented node was metastatic and as LNM-negative if no metastatic node was found in the specimen; no patient had metastatic disease confined to a node other than the one segmented. Only the ipsilateral neck was modelled. Extranodal extension was present in 11 of the 32 metastatic nodes. All patients underwent selective (levels I–III) or modified radical neck dissection, with a median of 28 nodes retrieved (range 18–46). The patient was the unit of prediction, data partitioning and performance reporting.

### 2.3. MRI Protocol

All patients were imaged on a 1.5 T scanner (Signa HDxt, GE Healthcare, Milwaukee, WI, USA) with a head and neck coil. The protocol comprised T1- and T2-weighted fast spin-echo images (repetition time [TR] 450 ms and 2500–3000 ms, respectively; echo time [TE] 10–70 ms; echo train length [ETL] 10; matrix 256 × 256 reconstructed to 512 × 512; slice thickness 3 mm; number of excitations [NEX] 2; field of view [FOV] 240 × 240 mm); axial diffusion-weighted single-shot spin-echo echo-planar images (TR 8400 ms, TE 17 ms, FOV 200 mm, matrix 120 × 120, slice thickness 3 mm, gap 0.3 mm); and contrast-enhanced T1-weighted images (CE-T1WI), acquired in the axial and coronal planes (TR/TE 550/10 ms, flip angle 90°, FOV 150 × 150 mm, matrix 320 × 256, slice thickness 3.5 mm, gap 0.3 mm, NEX 2) and as an additional thin-section coronal series (TR/TE1/TE2 6/2/3 ms, flip angle 15°, FOV 200 × 200 mm, matrix 192 × 190, slice thickness 1.1 mm).

### 2.4. Lymph Node Segmentation

One node per patient, the largest morphologically suspicious cervical lymph node, was manually segmented by an oral and maxillofacial radiologist (İ.Ö.), blinded to histopathology. The region of interest (ROI) was delineated with the freehand lasso tool of the RadCloud platform (Huiying Medical Technology Co., Ltd., Beijing, China) on every slice of the axial CE-T1WI series on which the node was visible, giving one volumetric ROI per patient. For the earlier slice-level analysis, the node had also been delineated on additional sequences, and slices from each sequence were counted separately, giving 1359 slices: 1284 from the 32 LNM-positive patients and 75 from the 42 LNM-negative patients. Twenty randomly selected nodes were re-segmented by the same reader after six weeks and independently by a second reader; the median intraclass correlation coefficient (ICC) of the extracted features was 0.87 (intra-reader) and 0.81 (inter-reader), and features with an inter-reader ICC below 0.75 were excluded.

### 2.5. Image Preprocessing

For the primary analysis, features were extracted from the axial CE-T1WI series only. N4ITK bias-field correction was applied, and images and masks were then resampled to 1 × 1 × 1 mm^3^ isotropic voxels using B-spline interpolation for the image and nearest-neighbour interpolation for the mask. Intensities were z-score normalised within the whole image, with values beyond three standard deviations clipped, and rescaled by a factor of 100. Grey levels were discretised with a fixed bin width of 5 (median 48 bins per ROI, range 31–66). Diffusion-weighted images and apparent diffusion coefficient maps were not used for feature extraction.

### 2.6. Radiomic Feature Extraction and Selection

A total of 1409 radiomic features were extracted with the RadCloud platform (v3.2, PyRadiomics 3.0.1): first-order, shape and texture features (grey-level co-occurrence matrix [GLCM], grey-level run-length matrix [GLRLM], grey-level size-zone matrix [GLSZM] and grey-level dependence matrix [GLDM]), computed from the original images and from exponential, logarithmic, square, square-root, gradient and eight three-dimensional wavelet-filtered images. Features were computed once from each volumetric ROI, giving one feature vector per patient.

Feature selection was performed inside each outer training fold. After ICC filtering, features were z-score standardised with training-fold parameters, near-constant features were removed, one feature of each pair correlated at |r| > 0.90 was discarded (retaining the member more strongly associated with outcome in the training fold), and the remainder were passed to least absolute shrinkage and selection operator (LASSO) logistic regression with the penalty tuned in the inner loop.

### 2.7. Model Development and Validation

Partitioning, feature selection, model fitting and evaluation were performed outside the RadCloud platform in Python 3.11 (scikit-learn 1.4, statsmodels 0.14). Six classifiers were compared: logistic regression (LR), random forest (RF), decision tree (DT), k-nearest neighbours (KNN), extreme gradient boosting (XGBoost) and support vector machine (SVM). Performance was estimated by repeated nested cross-validation with the patient as the unit of partitioning [20]: 5 outer folds stratified on LNM status, repeated 10 times with different random seeds, so that 14–15 patients were held out per fold and each patient was held out once per repeat. Within each outer training set, a 5-fold inner loop was used for feature selection, hyperparameter tuning by grid search and choice of the decision threshold (maximum Youden index). The averaged probability of each patient was classified using the mean of the fold-specific thresholds. Class imbalance (32:42) was handled with class-weighted loss functions where supported.

In each repeat every patient received one out-of-fold predicted probability; these were averaged across repeats, and the 74 resulting probabilities were used for all metrics: area under the receiver operating characteristic curve (AUC), precision–recall AUC (PR-AUC), sensitivity, specificity, positive and negative predictive values (PPV, NPV), balanced accuracy and overall accuracy, with the metastatic class as positive. Ninety-five percent confidence intervals (CIs) were obtained by percentile bootstrap over 2000 resamples of patients. Decision-curve analysis [21] was performed on the pooled out-of-fold probabilities.

### 2.8. Sensitivity Analysis: Slice-Level Design

To show the effect of the unit of analysis, the earlier slice-level analysis of the same data was retained as a sensitivity analysis. In that design each of the 1359 slices was treated as an independent sample, features were extracted per slice, 500 features were retained by an unsupervised variance threshold, and the slices were split into training (1087) and evaluation (272) sets, so that slices from the same patient could appear in both.

## 3. Results

### 3.1. Classifier Performance

**Figure 2:**
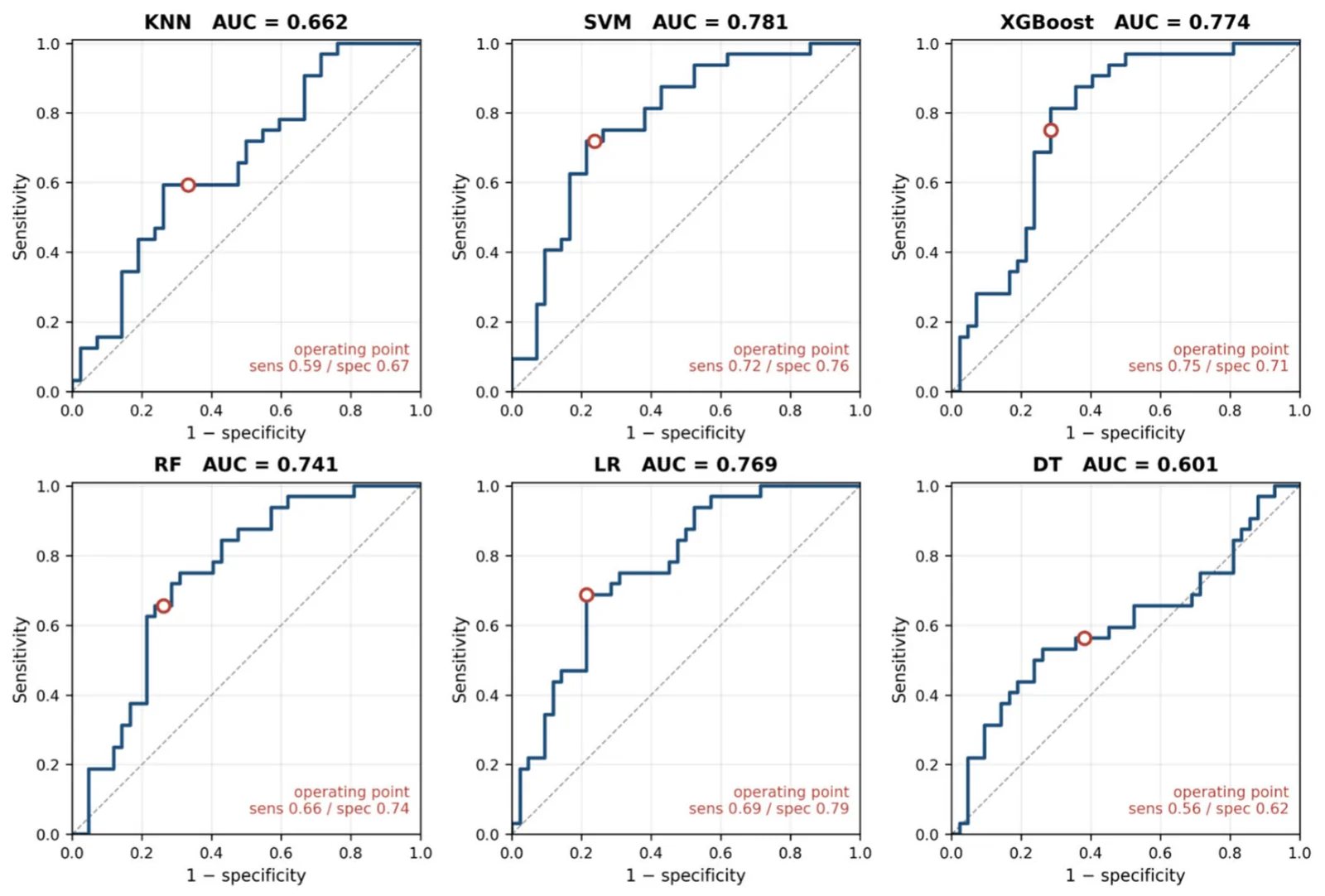
Receiver operating characteristic curves of the six classifiers from the pooled out-of-fold predictions of the 74 patients. The circle marks the operating point at the mean of the fold-specific thresholds. The dashed diagonal line indicates chance-level discrimination (AUC = 0.5). AUC: area under the receiver operating characteristic curve; sens: sensitivity; spec: specificity; KNN: k-nearest neighbours; SVM: support vector machine; XGBoost: extreme gradient boosting; RF: random forest; LR: logistic regression; DT: decision tree.

SVM gave the highest discrimination, with an AUC of 0.781 (95% CI 0.667–0.884), sensitivity of 0.72, specificity of 0.76 and balanced accuracy of 0.74 (Table 1, Figure 2). XGBoost (AUC 0.774, 95% CI 0.661–0.878) and logistic regression (AUC 0.769, 95% CI 0.655–0.874) performed comparably, whereas random forest, KNN and the decision tree performed less well (0.741, 0.662 and 0.601). The confidence intervals of the three leading models overlap almost completely, so their ranking should not be over-interpreted. Optimism, the difference between apparent and cross-validated AUC within the training folds, ranged from 0.09 for logistic regression to 0.38 for the decision tree (Table 2).

### 3.2. Classification Counts and Clinical Operating Points

At the threshold selected inside the training folds, SVM correctly classified 23 of the 32 LNM-positive and 32 of the 42 LNM-negative patients (Table 3), and its PR-AUC was 0.731 against a prevalence baseline of 0.432. Decision-curve analysis showed net benefit over treat-all and treat-none strategies for threshold probabilities of approximately 0.25–0.60. At a rule-out operating point with sensitivity 0.90, specificity was 0.48 and NPV 0.87.

### 3.3. Selected Features

A median of nine features (range 6–13) was retained across the 50 outer training folds. Seven features were selected in at least 80% of folds: skewness and 10th percentile (first order), cluster shade, correlation and inverse difference moment (GLCM), size-zone non-uniformity normalised (GLSZM) and sphericity (shape). The remaining selections varied between folds, so no single feature should be interpreted as biologically meaningful.

### 3.4. Comparison with the Slice-Level Analysis

The slice-level analysis gave an apparent AUC of 0.878 with a sensitivity of 0.97 but a specificity of only 0.33, and its overall accuracy of 0.93 was below the 0.945 that would be obtained by calling every slice metastatic (Table 4). At the patient level the corresponding majority-class baseline is 0.568.

## 4. Discussion

In this pilot study, radiomic features of the largest suspicious cervical lymph node on MRI discriminated LNM-positive from LNM-negative patients with moderate accuracy: the best model, SVM, reached a patient-level AUC of 0.781 (95% CI 0.667–0.884), and XGBoost and penalised logistic regression performed comparably. The confidence intervals are wide, and the present work is an exploratory step towards a preoperative decision-support tool rather than evidence that such a tool is achievable at this level of performance.

Relatively few studies have addressed radiomics for nodal staging in OSCC specifically. Lan et al. combined MRI radiomics with deep learning features in 319 patients with early-stage oral and oropharyngeal squamous cell carcinoma, using LASSO for feature selection and two external validation sets [22]; Wang et al. applied MRI-based radiomics and deep learning to tongue squamous cell carcinoma [23]; and Troise et al. evaluated machine learning for occult nodal metastasis in early-stage (cT1–T2/cN0) oral cavity carcinoma [24]. These studies share a patient-level unit of analysis, supervised and penalised feature selection and patient-independent validation, and their cohorts indicate the scale required; with 74 patients, the present study is a pilot. Our cohort included several oral subsites rather than the tongue alone, which may broaden applicability but also increases biological heterogeneity. Outside the head and neck, MRI-based radiomics or deep learning has been applied to nodal staging in endometrial [25] and rectal cancer [26], and CT radiomics with LASSO selection in gastric cancer [27]; the AUC of 0.892 reported by Xu et al. in endometrial cancer exceeds the 0.781 obtained here but came from a considerably larger cohort.

The slice-level analysis of the same patients (Table 4) illustrates two well-known pitfalls. First, slices from one node share anatomy, acquisition and segmentation boundaries, so under a slice-level split a test slice has near-duplicates in the training set and a model can recognise the patient rather than the disease. Second, because metastatic nodes are larger and yield more slices, a slice-level design creates a 17:1 class imbalance that does not exist at the patient level (32:42). The resulting model, with a sensitivity of 0.97 and a specificity of 0.33, reflected this imbalance rather than genuine discrimination. Because the two analyses also differ in feature dimension (500 features versus a median of 9), in two- versus three-dimensional extraction and in the resampling scheme, the difference between them reflects the earlier design as a whole rather than leakage alone.

The intended use determines the operating point. A model meant to identify patients who can safely avoid elective neck dissection is a rule-out test requiring high sensitivity and NPV, whereas a model meant to select patients for more extensive surgery is a rule-in test requiring high specificity and PPV. At a rule-out operating point, the NPV was 0.87, below the level needed to omit elective neck dissection, so the present model cannot yet serve that purpose.

### Limitations

This study has several limitations. First, 74 patients with 32 events support a pilot estimate only, and the confidence interval for the best model (0.667–0.884) ranges from marginal to good discrimination. Second, the design is retrospective, single-centre and based on a single 1.5 T scanner. Third, no external validation cohort was available; nested cross-validation controls optimism but does not replace evaluation in an independent population. Fourth, the model was not benchmarked against nodal size alone, against experienced head and neck radiologists or against clinical variables; because nodal size is encoded in the shape and first-order features of a volumetric ROI (sphericity was among the stable features), whether the radiomic signature adds information beyond size remains to be shown. Fifth, the cohort was not restricted to clinically node-negative necks, the setting in which prediction of occult metastasis matters most. Sixth, only the largest morphologically suspicious node was modelled, so metastatic disease confined to smaller nodes would not be captured. Finally, only seven features were selected consistently across folds, so the signature should not be interpreted biologically.

## 5. Conclusions

This pilot study developed MRI-based radiomics machine learning models for the preoperative prediction of cervical LNM in OSCC and validated them internally at patient level. SVM achieved a pooled out-of-fold AUC of 0.781 (95% CI 0.667–0.884), with sensitivity 0.72, specificity 0.76 and balanced accuracy 0.74; XGBoost and penalised logistic regression performed comparably. Performance is moderate and the confidence intervals are wide, so the models are not ready for clinical use. A slice-level analysis of the same data gave a higher apparent AUC but a specificity of only 0.33, showing how strongly slice-level designs can misrepresent performance. External validation in larger multicentre cohorts is the necessary next step.

## Figures and Tables

**Table 1:** Patient-level performance in the pooled out-of-fold predictions (*n* = 74; 32 LNM-positive, 42 LNM-negative).

Classifier	AUC (95% CI)	Sensitivity	Specificity	Balanced Accuracy	PPV/NPV
KNN	0.662 (0.535–0.782)	0.59	0.67	0.63	0.58/0.68
SVM	0.781 (0.667–0.884)	0.72	0.76	0.74	0.70/0.78
XGBoost	0.774 (0.661–0.878)	0.75	0.71	0.73	0.67/0.79
RF	0.741 (0.622–0.851)	0.66	0.74	0.70	0.66/0.74
LR	0.769 (0.655–0.874)	0.69	0.79	0.74	0.71/0.77
DT	0.601 (0.472–0.726)	0.56	0.62	0.59	0.53/0.65

Confidence intervals are percentile bootstrap intervals over 2000 resamples of patients. Sensitivity, specificity and predictive values refer to the metastatic class; balanced accuracy is the mean of sensitivity and specificity. AUC: area under the receiver operating characteristic curve; CI: confidence interval; PPV/NPV: positive/negative predictive value; KNN: k-nearest neighbours; SVM: support vector machine; XGBoost: extreme gradient boosting; RF: random forest; LR: logistic regression; DT: decision tree.

**Table 2:** Cross-validated and apparent AUC within the outer training folds, optimism, number of selected features and decision threshold.

Classifier	Inner-Loop CV AUC (Mean ± SD)	Apparent AUC in Training Folds	Optimism	Median Features Selected	Mean Youden Threshold
KNN	0.673 ± 0.071	0.88	0.21	9	0.44
SVM	0.796 ± 0.054	0.93	0.13	9	0.46
XGBoost	0.788 ± 0.061	0.99	0.20	9	0.48
RF	0.755 ± 0.066	0.99	0.24	9	0.47
LR	0.781 ± 0.049	0.87	0.09	9	0.45
DT	0.616 ± 0.083	1.00	0.38	9	0.50

Inner-loop CV AUC is the mean ± SD across the 50 outer training folds; apparent AUC is the AUC of each model on its own training data; optimism is the difference between the two. The Youden threshold is the mean of the thresholds selected in the inner loop. CV: cross-validation; SD: standard deviation; other abbreviations as in Table 1.

**Table 3:** Patient-level classification counts, overall accuracy and PR-AUC in the pooled out-of-fold predictions.

Classifier	TP (of 32)	FN	TN (of 42)	FP	Accuracy	PR-AUC
KNN	19	13	28	14	0.64	0.592
SVM	23	9	32	10	0.74	0.731
XGBoost	24	8	30	12	0.73	0.722
RF	21	11	31	11	0.70	0.681
LR	22	10	33	9	0.74	0.714
DT	18	14	26	16	0.59	0.521

Counts refer to patients (32 LNM-positive, 42 LNM-negative). The prevalence baseline for PR-AUC is 0.432. TP: true positive; FN: false negative; TN: true negative; FP: false positive; PR-AUC: precision–recall area under the curve.

**Table 4:** Slice-level versus patient-level analysis of the same 74 patients (SVM).

Aspect	Slice-Level Analysis	Patient-Level Analysis (Primary)
Unit of evaluation	272 slices	74 patients
Independence of evaluation data	Slices of the same patient in both sets	No patient in both sets
Features entering the model	500	Median 9 (range 6–13)
AUC	0.878	0.781
95% CI	0.75–1.00	0.667–0.884
Sensitivity	0.97	0.72
Specificity	0.33	0.76
Balanced accuracy	0.65	0.74
PPV/NPV	0.96/0.42	0.70/0.78
Overall accuracy	0.93	0.74
Accuracy of a trivial majority classifier	0.945	0.568

The slice-level column reproduces the earlier analysis, in which the 1359 slices were partitioned directly; the patient-level column gives the pooled out-of-fold results of the present pipeline. PPV/NPV: positive/negative predictive value. Other abbreviations as in Table 1.

## Data Availability

The anonymised radiomic feature data generated and analysed in this study are available from the corresponding author upon reasonable request. The shareable dataset comprises, for each of the 74 patients, an anonymised patient identifier, the histopathological class label, the 1409 volumetric radiomic features, the outer-fold assignment for each of the 10 repeats and the pooled out-of-fold predicted probabilities of each classifier. The underlying MR images cannot be shared publicly because of patient privacy and institutional restrictions.
